# Inherent α‐Helix Around Tyrosine 183 in the Intrinsically Disordered Tail of the Multi‐Site Docking Platform Gab1

**DOI:** 10.1002/bip.70120

**Published:** 2026-08-04

**Authors:** Anne Dietrich, Marc Lewitzky, Tobias Gruber, Jochen Balbach, Stephan M. Feller, Ulrich Weininger

**Affiliations:** ^1^ Tumor Biology Section, Institute of Molecular Medicine Martin Luther University Halle‐Wittenberg Halle (Saale) Germany; ^2^ Biophysics, Institute of Physics Martin Luther University Halle‐Wittenberg Halle (Saale) Germany; ^3^ Mitteldeutsches Zentrum für Struktur Und Dynamik der Proteine Martin Luther University Halle‐Wittenberg Halle (Saale) Germany

**Keywords:** α‐Helix, Gab1, intrinsic disorder, NMR, secondary structure, signal computation

## Abstract

The biological function of intrinsically disordered proteins is frequently coupled to short linear motifs, which serve as protein binding sites. This goes often hand in hand with local transient structural element formation. The intrinsically disordered C‐terminal region of the multi‐site docking protein Grb2‐associated binder 1 (Gab1) contains several well‐characterized phosphotyrosine pairs, whereas the Tyr162 and Tyr183 epitopes have remained practically unstudied. Here, we combine computational prediction, circular dichroism and high‐resolution NMR spectroscopy (chemical shift and relaxation analyses) to structurally characterize a Gab1 fragment containing residues 142–203. The here determined NMR structure shows an α‐helix for residues Pro180 to Ile187, with Tyr183 positioned centrally, while all other parts of the fragment, including the region around Tyr162, are disordered. This helical conformation is maintained upon changes in pH, variation of peptide lengths and phosphorylation status. The inherent helix around Tyr183 distinguishes the local structural environment of Tyr162 and Tyr183 within the intrinsically disordered Gab1 tail and provides a structural framework for future studies on interaction partners and potential functional roles of this structural motif.

## Graphical abstract

The two phosphorylation sites within the Gab1 fragment 142‐203 exhibit distinct structural characteristics. Tyr162 is in a disordered region, Y183 in a helical segment.

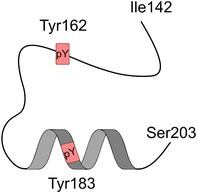

## 1 Introduction

Cellular signaling processes commonly rely on the formation of sophisticated, dynamic multi‐protein complexes and clusters to regulate vital biological functions [1, 2, 3]. In this context, large and mostly intrinsically disordered multi‐site docking platforms like the Grb2‐associated binder 1 (Gab1) protein have already emerged in simple metazoans like the demosponge *Amphimedon queenslandica* over 600 million years ago [4, 5, 6].

Gab1 lacks any known catalytic function but nevertheless mediates numerous crucial signaling processes in humans and other mammals, including the regulation of liver development and regeneration, as well as muscle precursor cell migration into the extremities during embryogenesis [7].

**FIGURE 1:**
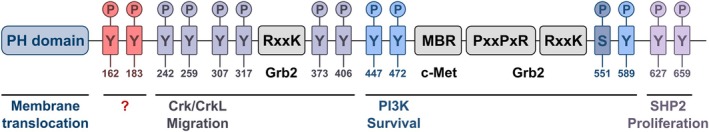
Schematic depiction of domains, interaction sites and phosphotyrosine pairs in Gab1. The N‐terminal pleckstrin homology (PH) domain (aa 1–125) mediates membrane translocation. The intrinsically disordered tail contains multiple functional pairs of phosphorylation‐regulated tyrosines. Other protein interaction sites for Grb2 SH3 domains (RxxKP, PxxPxR) and the tyrosine kinase receptor c‐Met (MBR) are also indicated. The serine 551 epitope regulates the functionality of the PH domain in a phosphorylation‐dependent manner. Central Tyr‐xxx‐xxx‐Pro (YxxP) motifs bind to Crk/CrkL and Nck adaptors to control cell migration when phosphorylated, while the more C‐terminal Tyr‐xxx‐xxx‐Met (YxxM) motifs promote cell survival and the two most distal tyrosines bind SHP2, which affects MAPK pathway signaling and proliferation. In contrast to these well‐studied modules, the two putative phosphotyrosines Tyr162 and Tyr183 remained virtually uncharacterized.

Gab1 consists of a ca. 120 amino acid long N‐terminal pleckstrin homology (PH) domain involved in phospholipid binding and translocation of Gab1 to cell membranes, followed by a large intrinsically disordered (ID) tail region of ca. 570 amino acids important for the assembly of specific signaling protein complexes (Figure 1). This high degree of intrinsic disorder is typical for many signal “computation” platforms or hub proteins in cells that integrate cellular signals [8]. A similar protein organization is also found, for example, in the members of the IRS, p130Cas/BCAR1 and FRS protein families, which echo the Gab1 protein composition, that is, display folded domains N‐terminally, followed by long intrinsically disordered tails that encompass multiple phosphorylatable tyrosines [5, 9]. Gab1 is virtually ubiquitously expressed in human cells and a target of multiple tyrosine kinases. Thus, it can become phosphorylated on numerous distinct sites, depending on cell type and biological context. Deregulation of these phosphorylation events can lead to a wide range of diseases, including numerous cancers [10].

The multiple tyrosine phosphorylations within the Gab1 protein create docking sites for several protein interaction partners containing Src homology 2 (SH2) domains. Sequence analyses and functional studies of the Gab1 tyrosine phosphorylation sites have shown that these docking sites are not randomly distributed throughout the long ID tail, but appear paired (Figure 1), revealing a certain degree of architectural principle. Between Gab1 amino acids 242 and 410, no less than 6 Tyr‐xxx‐xxx‐Pro motifs can be found. Upon phosphorylation, Gab1 has been shown to bind SH2 domain proteins like Crk family adaptors, the adaptor protein Nck, and the phospholipase PLCγ [11, 12, 13]. The docking of Crk and Nck adaptors has been linked to specific biological actions, including cell migration and membrane ruffling [14]. More C‐terminally, three phosphorylatable Tyr‐xxx‐xxx‐Met motifs are located, which have been shown to interact with the SH2 domains of PI3 kinase, a cell survival regulator [4]. The two most C‐terminal tyrosines, Tyr 627 and Tyr 659, are both well‐characterized docking sites for the tandem SH2 domains of the protein tyrosine phosphatase SHP2, a regulator of the Ras GTPase and its modulators [15, 16], mediating the activation of the Ras–MAPK signaling pathway and driving proliferation [17]. The last tyrosine of Gab1, Tyr 659, is unusual in that it is located near a transient short α‐helix in the Gab1 ID tail otherwise largely void of recognizable secondary structure that is involved in SHP2 SH2 domain binding by an encounter complex even in the absence of phosphorylation [18, 19].

**FIGURE 2:**
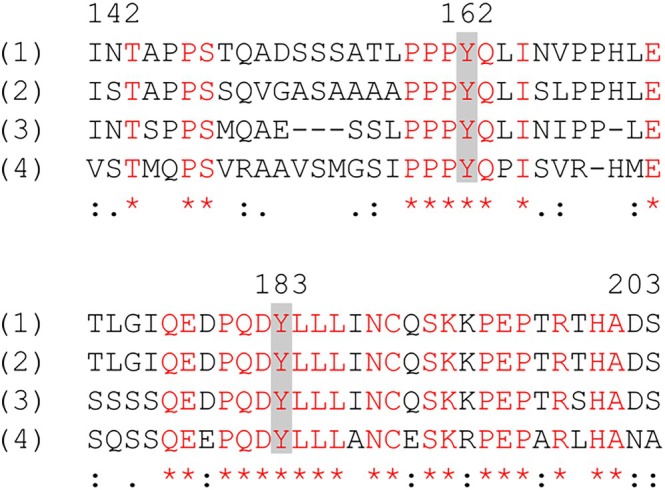
A highly conserved region following the Gab1 PH domain contains Y162 and Y183. Alignment of the investigated protein region of (1) *Homo sapiens* , (2) *Bos taurus* , (3) *Gallus gallus*, and (4) *Callorhinchus milli*. The bottom line indicates the positional conservations, where * and red color mark a fully conserved residue across all species, : shows strong similarity and . indicates a weaker similarity. The alignment illustrates a high degree of evolutionary conservation in the vicinity of the two tyrosines Tyr162 and Tyr183.

Another tyrosine, Tyr 183, is located N‐terminally of the Tyr‐xxx‐xxx‐Pro cluster, evolutionarily highly conserved (Figure 2) and repeatedly reported to be phosphorylated in mass spectrometry‐based cell studies according to PhosphoSitePlus [20]. Data from pTyr‐peptide library screens indicate that phosphorylated Tyr 183 could be a target site for several SH2 domains [21], putatively adding to the already significant binding partner portfolio of Gab1. The only other tyrosine nearby Tyr 183 and outside of the PH domain, namely Tyr 162, shares superficial similarity with the Tyr 183 epitope in that a cluster of three identical residues is located directly next to it, but was not reported to be phosphorylated so far. Furthermore, the Pro‐Pro‐Pro‐Tyr motif in the Tyr 162 epitope, in its unphosphorylated form, could potentially serve as a binding site for group I WW domains, which preferentially bind to Pro‐Pro‐xxx‐Tyr motifs [22, 23] raising the question if the Tyr 162 epitope is possibly multifunctional. Taken together, these features of the Tyr 162 and Tyr 183 epitopes in Gab1 encouraged us to embark on biophysical analyses of this still enigmatic region in the large, intrinsically disordered multi‐site docking platform Gab1, in order to determine if secondary structural elements that may be functionally important are detectable.

Intrinsically disordered regions (IDR) or proteins (IDP) are suitable targets for NMR spectroscopy, which can detect transient local structures, known as “prestructured motifs” or “preformed structures” within dynamically plastic regions [24, 25]. Here we shed light on two conserved Gab1 tyrosines by thoroughly investigating a peptide fragment which includes Y162 and Y183 and adequate N‐ and C‐terminal extensions, namely Gab1 Ile142‐Ser203. CD spectroscopy shows some small overall α‐helical propensity in the fragment, while an NMR structure pinpoints the α‐helix content to residues 180–187 including Y183, while no local structures are detectable in other parts of the peptide fragment, including Tyr 162. Ultimately, both phosphorylation sites are distinguished by structure. This α‐helix persists across changes in pH, peptide length, and phosphorylation status.

## 2 Materials and Methods

### 2.1 Cloning

In order to generate the construct for the recombinant protein expression of SUMO‐Gab1 (Ile142‐Ser203), vector backbone and insert were amplified separately by PCR using specifically generated forward and reverse primers. The backbone was amplified from pET‐His‐SUMO‐hGab1 for the amino acids of Gab1 (142‐203) and the insert from pET‐His‐SUMO‐SHP2‐SH2C for the SUMO‐Tag. Both were analyzed on a 1% agarose gel and then purified. Backbone and insert were assembled using Circular Polymerase Extension Cloning (CPEC) and the resulting plasmid was then transformed into the competent cell line *E. coli* XL10‐gold. After isolating the DNA by miniprep (miniprep kit, Qiagen) and sequence verification (Microsynth), the plasmid was transformed into *E. coli* Bl21 (DE3) for protein expression.

### 2.2 Expression and Purification

The transformed *E. coli* BL21 (DE3) were cultured either in Terrific Broth (TB) or M9 minimal medium, which has exclusively ^13^C‐glucose and ^15^N‐ammoniumchloride as carbon and nitrogen sources, for unlabeled or uniformly ^15^N^13^C labeled samples, respectively.

The culture was incubated at 37°C until an OD600 of 1.6 was reached and then expression induced with 0.5 mM IPTG. cultivation continued overnight (16 h) at 18°C. Cells were harvested by centrifugation at 3000×*g* in a precooled centrifuge (J6‐MI) for 30 min. Cell pellets were resuspended in 50 mM NaCl, 50 mM Tris–HCl pH 7.5 and lysed by sonication (2 × 1 min) on ice. Lysate was centrifuged for 60 min at 30,000×*g* (Avanti JXN‐26) and the supernatant again for 120 min at 60,000×*g* (Avanti JXN‐26).

Clarified lysate was applied to a HiTrap Column (IMAC FF, Ni^2+^). Bound protein was eluted with imidazole and its concentration measured using UV‐spectroscopy at 280 nm. SUMO protease was added (1:50) and the mixture dialyzed in a 3.5 kDa cut‐off dialysis tube in 2 L dialysis buffer (150 mM NaCl, 50 mM Tris–HCl pH 7.5) for 3 h at 4°C and then in fresh 2 L dialysis buffer overnight (16 h) at 4°C. After cleavage of the SUMO‐tag, the sample was re‐applied onto a HiTrap Column (IMAC FF, Ni^2+^). The flow‐through containing the protein was collected, while the SUMO‐tag and SUMO‐protease bound to the column.

For final purification and buffer determination, the sample was applied to a size exclusion chromatography (Superdex pg. 75, Cytiva). Concentration was adjusted with a Vivaspin 5 kDa MWCO concentrator.

### 2.3 Synthetic Phosphopeptides

Phosphorylated 19 amino acid peptides with C‐terminal amidation were purchased from ProteoGenix (France) as lyophilized, HPLC‐purified products (> 98% purity, TFA‐free). In addition, a synthetically double phosphorylated Gab1 peptide (residues Thr157‐Lys192), containing phosphorylated Tyr162 and Tyr183, was purchased as a lyophilized, HPLC‐purified product (> 98% purity, TFA‐free). Peptides were dissolved in appropriate buffer before use.

### 2.4 Disorder Prediction (IUPred3) and Missense Variant Prediction (AlphaMissense)

Intrinsic disorder was predicted with the short disorder mode of IUPred3 (iupred3.elte.hu) and residue‐level disorder scores were extracted for further analysis. AlphaMissense (alphamissense.hegelab.org) was used to evaluate the potential effects of point mutations and also predicted on the official website. Data were downloaded and processed with Excel.

### 2.5 Circular Dichroism (CD) Spectroscopy

Far‐UV CD spectra were measured on a Jasco J‐1500 spectropolarimeter at room temperature (22°C) in a 1 mm quartz cuvette with a protein concentration of 1 mg/mL in 150 mM NaCl, 50 mM Tris–HCl pH 7.5. Chloride‐containing buffer was chosen to stabilize the peptide solution and to maintain buffer consistency. Spectra were measured from 190 to 260 nm in 0.2 nm steps and accumulated 25 times. For TFE titration experiments, spectra were recorded under identical conditions in the presences of increasing concentrations (0%–90%) of 2,2,2‐trifluoroethanol (TFE). Raw ellipticity values (in mdeg) were converted to the mean residual ellipticity (MRW, deg. × cm^2^ × dmol^−1^) with the following equation to allow direct comparison independent of concentration and length of protein chain.

$$\mathrm{MRW}=\frac{\theta }{c\times l\times N\mathrm{aa}\times 10}$$

with

*θ* = ellipticity (mdeg).

*c* = molar concentration (M).

*l* = path length (cm).

*N*aa = number of amino acids.

### 2.6 NMR Spectroscopy

NMR experiments were performed on a Bruker Avance III 800 MHz‐NMR spectrometer with a TCI‐CyroProbe on a 1 mM ^15^N^13^C sample in 50 mM NaCl, 20 mM Bis‐Tris pH 6.5, with 10% (v/v) D_2_O at 25°C, unless indicated otherwise. The 2D ^1^H^15^N HSQC and aliphatic constant‐time ^1^H^13^C HSQC were acquired with 10,416 × 1865 Hz/12,820 × 14,085 Hz, 192/256 complex points in the indirect dimension and 16/32 scans. Resonances were assigned using standard 3D HNCACB, HN(CO)CACB, H(C)CH‐TOCSY (15.5 ms mixing) as well as 3D TOCSY‐HSQC (60 ms mixing) experiments for ^15^N nuclei and 3D NOESY‐HSQC (300 ms mixing) experiments for ^15^N and ^13^C aliphatic/aromatic nuclei. All NMR data were processed using NMRPipe and with NMRView. Chemical shift assignments have been uploaded to the BMRB (bmrb.io) under the entry number 52691. Structures were calculated using ARIA2.3 with standard parameters, with NOEs from the 3D NOESY‐HSQC experiments and Phi–Psi dihedral angle constraints derived using TALOS. The α‐helical content at different pH values and of shorter or phosphorylated constructs was monitored by aliphatic ^1^H^13^C‐HSQCs of about 3 mM samples with ^13^C at natural abundance.

Chemical shift indices were calculated by comparing experimental *C*_*α*_ and *C*_*β*_ with random coil reference values [26] using $\mathrm{CSI}=\left(C_{\alpha }-C_{\beta }\right)\;\exp-\left(C_{\alpha }-C_{\beta }\right)\;\mathrm{ref}$. While positive values indicate an α‐helical tendency, negative suggest an extended conformation.

^15^N relaxation experiments were conducted on a Bruker Avance III 600 MHz‐NMR spectrometer with a TXI room temperature probe using relaxation delays of 0, 50, 100, 200, 400, 700, and 1000 ms for *R*_1_, 0, 8, 16, 48, 80, 112, and 160 ms for *R*_2_, and a recycle delay and saturation period of 8 s and 3 s for the {^1^H‐}^15^N NOE.

## 3 Results and Discussion

To examine the regions surrounding Y162 and Y183 in Gab1, we used a construct spanning residues I142 to S203, which includes both tyrosines as well as N‐ and C‐terminal flanking regions of 20 amino acids.

### 3.1 Computational Preanalysis

**FIGURE 3:**
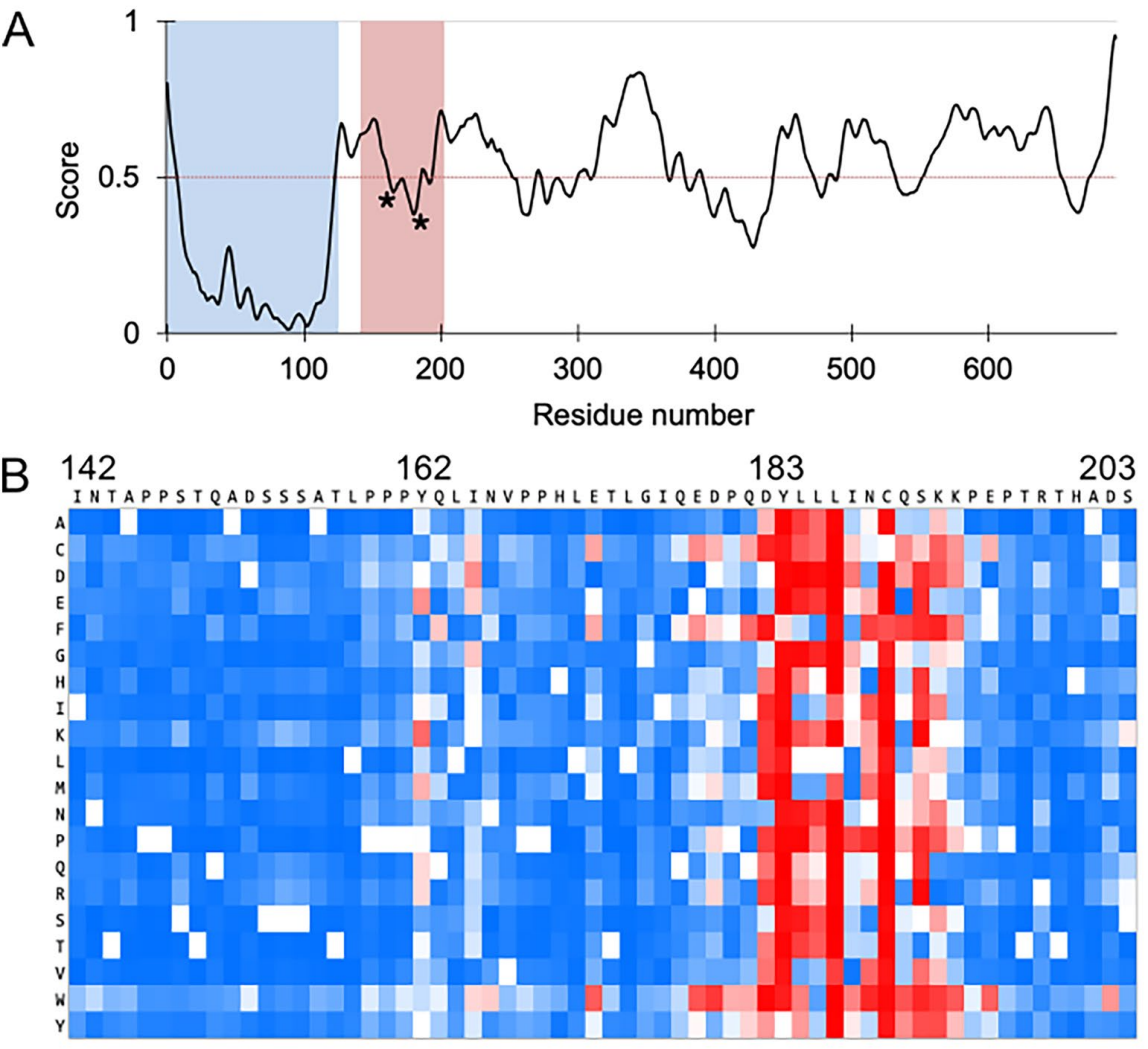
Disorder Prediction and AlphaMissense pathogenicity heatmap of Gab1. (A) IUPred 3 (short) prediction of full length Gab1 highlighting regions with reduced disorder propensity. The structured PH domain (amino acid Met1‐Val125) is shown in light blue, reflecting its known structure (PDB ID: 1F5Q). The here studied disordered region I142‐S203 is shown in light red and predicted to have somewhat reduced intrinsic disorder around Tyr162 and Tyr183. (B) AlphaMissense prediction of Grb2‐associated‐binding protein 1 (Gab1) from amino acids I142 to S203 shows presumably benign (blue) and pathogenic (red) mutations, where each cell represents a single missense variant with each residue position exchanged against one of the 20 natural amino acids.

In order to identify regions of potential functional or structural importance we first analyzed the whole Gab1 protein with IUPred 3 [27], followed by an analysis of the here studied region of interest with AlphaMissense [28]. The IUPred3 (short) predicts the likelihood of each residue to be disordered providing a score from zero (no tendency to be disordered) to one (very high possibility to be disordered), with 0.5 as a cutoff. The IUPred3 output of the full‐length Gab1 protein (Figure 3a) revealed the expected well‐structured N‐terminal region corresponding to the PH domain (residues Met1‐Val125), while the C‐terminal part is predicted to be largely unstructured, confirming earlier findings [19, 29]. Interestingly, around the two tyrosine residues Tyr162 and especially Tyr183 there is some secondary structure potential shown, suggesting a locally ordered preference instead of an overall disorder. This goes along with the evolutionary conservation of this region. The AlphaFold model of human Gab1 predicts structural differences between the two tyrosine epitopes. While the region around Tyr162 is predicted with low confidence (pLDDT ~40), which is consistent with intrinsic disorder, the segment surrounding Tyr183 displays a local helical conformation with moderately increased confidence values (pLDDT ~70) (Figure S1). Although the confidence values remain below those typically observed for well‐folded proteins, the prediction is consistent with a locally preferred structural element embedded within an otherwise intrinsically disordered region. We further explored this region of the two tyrosines (I142‐S203) with AlphaMissense, a prediction tool based on Alphafold2, classifying single amino acid mutations as either benign (blue), pathogenic (red) or uncertain (gray) (Figure 3b and Figure S2). Most of the mapped region is blue, suggesting a tolerance to mutations. While for Tyr162 and close residues a low pathogenicity was predicted, the residues Asp182 to Lys193 including Y183 appear mostly in red, documenting that mutations in this segment are classified as presumably pathogenic. While these findings have to be treated with care, they point to a potentially interesting epitope.

### 3.2 Biophysical Characterization

Given the potential functional importance and structural particularity of this region, we examined the structural properties of Gab1 I142‐S203 experimentally by circular dichroism (CD) and nuclear magnetic resonance (NMR) spectroscopy.

**FIGURE 4:**
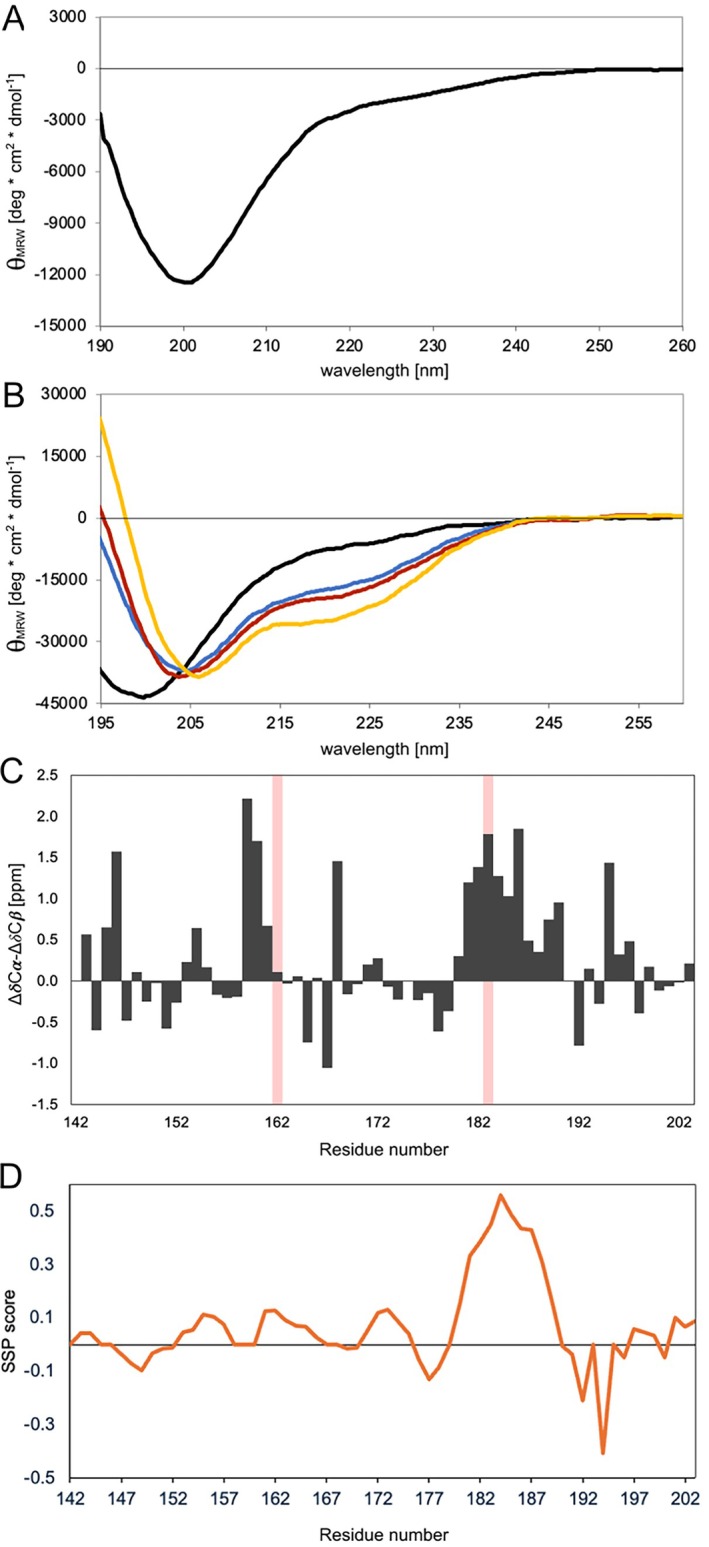
Circular dichroism spectra and NMR chemical shift analysis of Gab1 I142‐S203. (A) Circular dichroism spectrum of Gab1 I142‐S203 showing an overall disordered signal, with a minor negative ellipticity at 220 nm as indication of a presumably small α‐helical content. (B) Far‐UV CD spectra of Gab1 I142‐S203 were recorded in the absence (black) and presence of increasing concentrations of 2,2,2‐trifluoroethanol with blue (30%), red (60%), and yellow (90%) of TFE. Increasing TFE concentrations shifted the spectrum toward a characteristic α‐helical shape of the curve. (C) Bar plot of *C*_*α*_ and *C*_*β*_ chemical shift deviations from random coil values with Tyr162 and Tyr183 highlighted in light red. Positive values of Δ*C*_*α*_—Δ*C*_*β*_ above about 1 are indicative of α‐helical structures, negative values below about −1 for β‐strand or extended conformations. (D) Residue‐resolved SSP analysis with SSP scores calculated from C_α_, *C*_*β*_, and H_α_ chemical shifts. A continuous positive helical propensity is shown for the Pro180‐Ile187 segment.

The CD spectrum (Figure 4A) reveals a distinct minimum at around 200 nm, while there is only a weak negative dent around 220 nm. This signal pattern corresponds to a mostly intrinsically disordered peptide, respectively a random coil. Characteristic patterns for α‐helical or β‐sheet conformations are not detectable, except for the shallow minimum around 220 nm, indicating some low helical content. The data were subsequently analyzed for the amount of secondary structure with BeStSel, K2D2, and K2D3 [30, 31]. BeStSel, which is mainly based on large experimental data sets from clearly defined proteins, estimates 6% helical content. K2D2 and K2D3 predicted 8% and 2% of helical content, respectively. These analysis tools are optimized for folded proteins. In addition, the here used salt concentration is not optimal for CD spectroscopy. Consequently, all methods are somewhat limited and gave a different outcome. However, there is a clear tendency to have at least a small amount of helical content.

To further assess whether the observed helical content reflects an intrinsic structural propensity of the sequence, CD spectra were additionally recorded in the presence of increasing concentrations of 2,2,2‐trifluoroethanol (TFE). This shifted the spectrum progressively toward a characteristic α‐helical shape, with the minimum near 200 nm moving toward 208–210 nm and the appearance of a second minimum around 222 nm (Figure 4B). These spectral changes demonstrate that the Gab1 fragment Ile142 to Ser203 has an intrinsic propensity to adopt an α‐helical conformation under helix‐promoting conditions. Similar behavior has been observed for the Gab1 fragment 613–694 [19].

In order to determine the secondary structure content on the level of individual amino acids, we conducted NMR spectroscopy. The backbone was assigned by HNCACB‐ and HN(CO)CACB‐experiments (Figure S3). The assigned chemical shifts of *C*_*α*_ and *C*_*β*_ were then used to analyze the secondary structure content (Figure 4C) as previously described by calculating the chemical shift deviations Δ*C*_*α*_ and Δ*C*_*β*_ from random coil values [32]. Positive Δ*C*_*α*_—Δ*C*_*β*_ values indicate α‐helical conformations, while negative values indicate β‐strand or extended conformations [33]. Throughout most parts of the analyzed peptide the residues only show slight deviations from zero, with no consistent trend over several residues, which is compatible with the absence of a stable secondary structure. However, two regions show a clear difference. The first region around the residues of Pro159, Pro160, Pro161, and Tyr162, with their positive values, indicates a local structural propensity. Given the proline‐rich sequence, in addition to how short it is, this region is unlikely to adopt an α‐helix, it could be some transient polyproline II conformation, but there is no direct evidence for it. A larger, second region is located between Asp179 and Cys189, showing consecutively positive values. This indicates a local structural preference for a helical structure for these 11 residues around Tyr183 in the otherwise disordered peptide. To complement the conventional chemical‐shift analysis with an approach developed for intrinsically disordered proteins, the *C*_*α*_, *C*_*β*_, and *H*_*α*_ chemical shifts were analyzed using the SSP algorithm [34]. The resulting profile remained close to zero throughout most of the Gab1 fragment, consistent with a predominantly disordered ensemble (Figure 4D). In contrast, residues Pro180‐Ile187 formed a continuous positive segment with a mean SSP score of approximately 0.40, supporting a substantially populated local α‐helical conformation around Tyr183. The Tyr162 region displayed only weak positive values and no comparable continuous α‐helical propensity.

Both regions around Tyr 162 and Tyr 183 show further deviating NMR properties from a fully IDR. The amino acids *C*_*α*_ position show reduced intensities in a 2D ct ^1^H^13^C HSQC spectrum (Figure S4) and the ^15^N relaxation data (Figure S5) revealed increased *R*_2_ (and to a lesser extent *R*_1_) rate constants and slightly positive {^1^H‐} ^15^N NOE values (only around Y183). Together, all NMR properties are in agreement with an α‐helical propensity around Tyr183 and some loss of flexibility around Tyr162.

### 3.3 NMR Structure Determination

For a detailed structural characterization of the Gab1 fragment Ile142 to Ser203, we assigned additionally to the backbone all side chains NMR resonances and calculated the NMR structure in solution with the program ARIA [35], based on NOEs and dihedral restraints from chemical shifts using TALOS [36] (Table S1).

**FIGURE 5:**
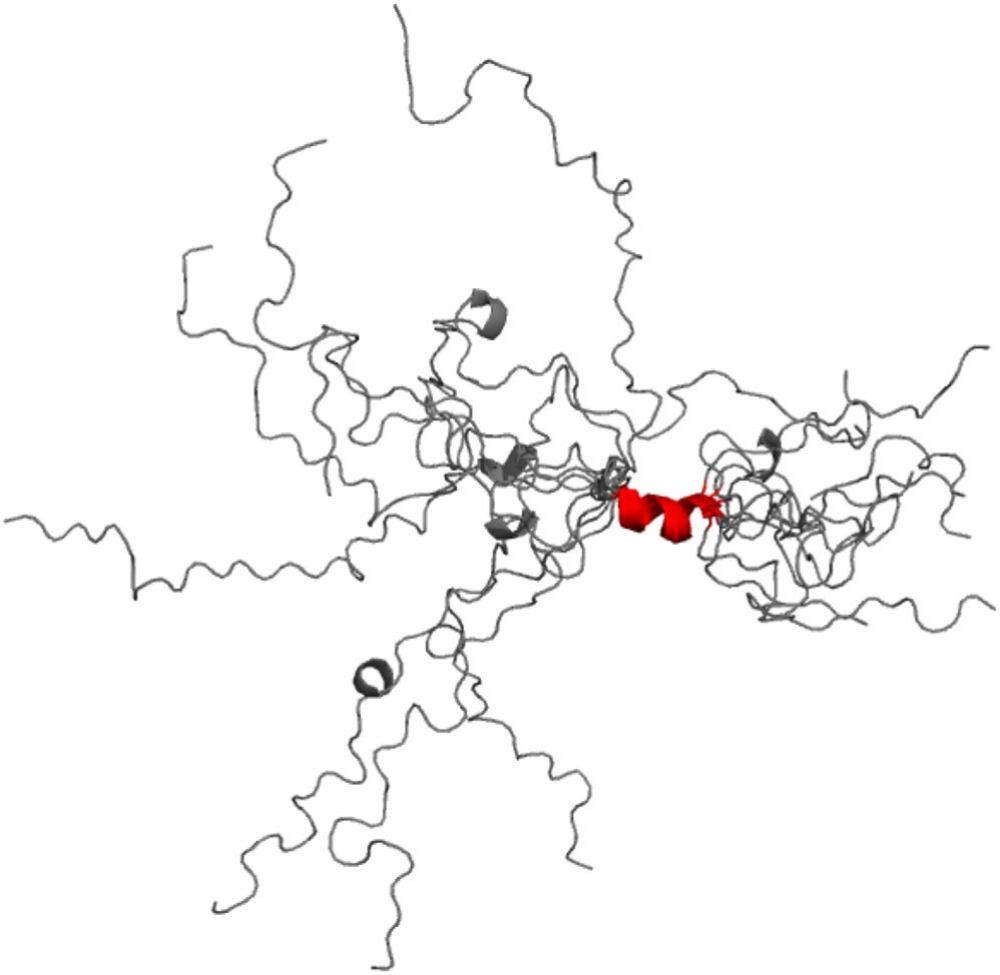
NMR structure of Gab1 Ile142‐Ser203 in solution. Overlay of the 10 lowest energy structures of Gab1 fragment I142‐S203, aligned for Pro180 to Ile187. The backbone structures are shown in ribbon representation. The eight‐amino‐acids‐long helical structure is shown in red.

The analysis of the derived structure models converges remarkably on one local section containing Pro180 to Ile187, which is supported by 20 residue (i) to residue (i‐3) NOEs in this region. These eight amino acids—containing Tyr183 in its middle—show in all 10 lowest energy models a well‐defined α‐helical structure. Since one complete turn of a helix spans approximately 3.6 amino acids, the secondary structure encompasses more than two turns. The convergence of 10 independently calculated backbone conformations and the dispersion of backbone angles in the Ramachandran plot document that this helical segment is the only robust structural element in these mainly disordered protein fragments (Figures 5 and S6). Outside of this helical structure, all models show a broad distribution of possible conformations, which are typical for intrinsically disordered peptides. This includes the region around Tyr162. However, in some models there is some tendency toward a helical structure albeit not as prominent as the helix around Tyr183, both in terms of structure and in terms of the underlying NMR restraints. The distinct structural behavior of the Tyr162 and Tyr183 regions may be explained by differences in their local sequence composition. While the Tyr162 region is enriched in proline residues, the Tyr183 region contains several leucine residues, which can stabilize an α‐helix [37].

It is important to note that, although the NMR‐derived ensemble reveals a well‐defined helix, this element is not necessarily populated in every molecule at all times. From the CD data, the overall α‐helical content of the 62‐residue fragment is ~5% (depending on the analysis method). Assuming an eight‐residue helix, this corresponds to an estimated average helix population on the order of ~40%. NMR structure calculations are intrinsically biased toward ordered conformations because the dominant NOE restraints arise primarily from the structured subpopulation; molecules lacking the helix contribute far fewer strong distance restraints and are therefore underrepresented in the resulting models. Thus, the most appropriate interpretation is a dynamic equilibrium in which the helix is present in a substantial fraction of molecules, roughly estimated on the order of about half (~40%), and absent in the remainder.

### 3.4 Assessment of the Helix Under Different Conditions

To assess if this helix is sensitive to pH changes, Gab1 I142‐S203 was analysed at pH 6.5 (conditions of the structure calculation), pH 7.0 and pH 7.4 regarding their ^13^ *C*_*α*_ carbon chemical shift, focussing on the eight amino acids contained in the helical structure.

**FIGURE 6:**
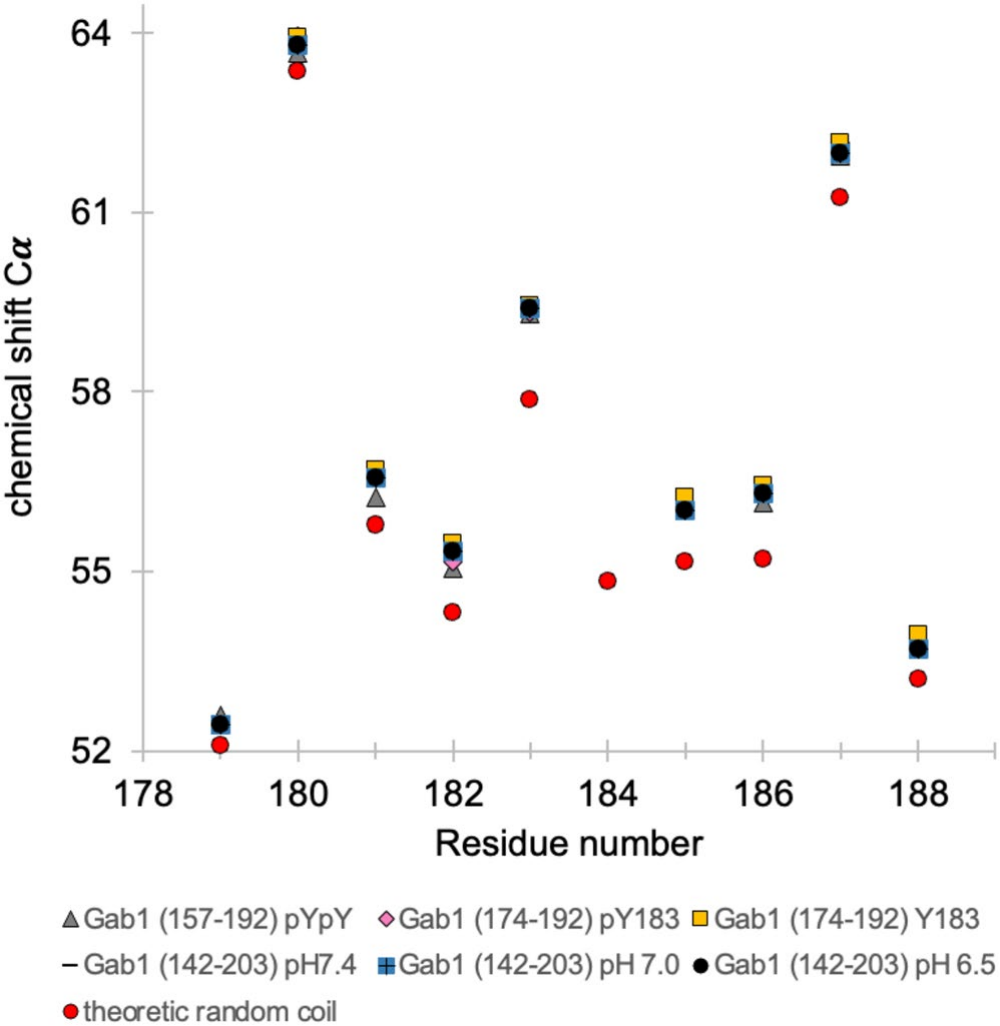
Consistent ^13^ *C*_*α*_ shifts confirm condition‐independent helix formation in Gab1 I142‐S203. ^13^ *C*_*α*_ chemical shifts of the residues 179–188 (helix 180–187 ± 1 residue) in Gab1 for a theoretically random coil (red) and measured for Gab1 (142–203) at pH 6.5 (black), 7.0 (dark blue), and 7.4 (light blue). Also compared across Gab1 peptides of different lengths (Gab1 (174–192) Y183 in yellow) and phosphorylation states (Gab1 (174–192) pY183 in pink and Gab1 (157–192) pYpY in gray). The highly overlapping values indicate that helix formation in this region is largely unaffected by pH, peptide length, or phosphorylation at Y162 or Y183.

The strong overlap of the observed values shows that the helical structure is maintained under different pH conditions (Figure 6). Experimental values for residue 184 could not be extracted reliably due to spectral overlap and are therefore not included in the analysis.

A further comparison was done by analysing a shorter peptide (Leu174 to Lys192), in order to test for potential long‐range effects. Also, here the chemical shift of each residue is nearly identical (Figure 6). The same holds if Tyr 183 is phosphorylated, which is reported according to PhosphositePlus in several cellular contexts, in the Leu174 to Lys192 construct, as well as with double phosphorylation (Y162 & Y183) in a Thr157 to Lys192 construct.

In summary we can conclude that the helix is present to about the same degree under different physiologically relevant conditions. Therefore, one can assume a helix (180–187) in Gab1, which is persistently present in half of the molecules.

### 3.5 Comparison With Other Structural Motifs in Gab1

Although the NMR structure reveals a clearly defined α‐helix, overall, the region shows a typical signature of an intrinsically disordered protein (IDP). Furthermore, a significant moiety in the ensemble of conformations of these eight residues is disordered according to the CD spectra and the Δ*C*_*α*_—Δ*C*_*β*_ NMR chemical shift deviations only reaching half maximum values. Thus, the helix represents a local structure in dynamic equilibrium within a largely intrinsically disordered conformational ensemble.

Consistent with the distinct structural environments of Tyr162 and Tyr183 this local α‐helix around Tyr183 may contribute to differences in molecular recognition or accessibility within this ID region of the hub protein Gab1. While the Tyr183 is located in a preorganized structural element, the surrounding region of Tyr162 remains largely disordered. Since phosphorylation of neither Tyr162 nor Tyr183 measurably change these structural properties, our data suggests that phosphorylation is not involved in helix formation or disruption as observed in other proteins. Instead, structure and phosphorylation may represent two complementary regulatory layers. The α‐helix appears to be an intrinsic property of the local sequence, while the phosphorylation may primarily function as a modular recognition signal for proteins targeting phosphorylated epitopes.

Phosphorylation does not induce helix formation. This differs from the mechanism described for other signaling proteins, where phosphorylation within disordered regions can trigger helix formation and with this enables effector binding. For example, the phosphorylation of a tyrosine residue in the IDR of TACC3 induces helix formation that allows binding to clathrin heavy chain (CHC) [38]. The Tyr162 segment does not display phosphorylation‐dependent helix formation and remains predominantly disordered. The situation for the Tyr183 segment is more complex. The partially preformed helix could become a full helix or a fully extended conformation upon binding. Both the isolated Gab1 (613–694) region and the complex with the tandem SHP2 SH2 domains adopt mostly extended conformations independently of the phosphorylation state [18, 19]. Similarly, available structures of SH2 domain–phosphotyrosine peptide complexes from the PDB (such as entries 1TCE and 1OO4) frequently display extended peptide conformations. The presence of the local helix around Tyr183 raises the possibility that this region may differ structurally from many canonical extended phosphotyrosine motifs. However, interactions with SH2 domains cannot be excluded, since several SH2–phosphotyrosine interactions involving helical or partially helical peptide segments have been reported [39, 40, 41].

Furthermore, a previously described complex of Grb2 with Gab1 (497–528) shows an extended polyproline II‐helix, which is analogous to other SH3‐mediated complexes such as CD2AP_2:RIN, Fyn:App12, and p40phox:p47phox [42]. In this context, the described transient α‐helix around Tyr183 represents a distinct structural state that may preorganize this fragment and thereby modulate accessibility and binding specificity.

## 4 Conclusion

Our structural analyses of the Gab1 region, which encompasses Tyr162 and Tyr183, reveal a previously unknown helix (P180 to I187) around Tyr183 within the otherwise intrinsically disordered tail of this important signaling protein. The residues constituting this helical element are evolutionarily conserved and show a striking robustness to moderate changes in pH, variations, peptide length, and phosphorylation. This argues that the Tyr183‐centered helix is an inherent property of the sequence rather than a condition‐specific artifact.

Taken together, these results reveal distinct structural environments for the two tyrosines and provide a structural framework for future studies investigating the potential functional relevance of this asymmetry. In addition, these findings extend the framework for understanding how vicinal tyrosine motifs within an intrinsically disordered region can profoundly differ in their conformational properties.

## Author Contributions

**Anne Dietrich:** designed research, performed experiments, analyzed data, wrote manuscript. **Marc Lewitzky:** designed research, performed experiments. **Tobias Gruber:** performed experiments. **Jochen Balbach:** designed research, analyzed data, wrote manuscript. **Stephan M. Feller:** designed research, wrote manuscript. **Ulrich Weininger:** designed research, analyzed data, wrote manuscript.

## Funding

This work was supported by Deutsche Forschungsgemeinschaft (391498659), European Regional Development Fund (2.21.6.05.00056, ZS/2016/04/78115), and the Bundesministerium für Bildung und Forschung (03Z22HN22).

## Conflicts of Interest

The authors declare no conflicts of interest.

## Supporting information


**Table S1:** Statistics of the NMR structure determination.
**Figure S1:** AlphaFold prediction of the human Grb2‐associated‐binding protein 1 (Gab1) for the region of Ile142‐Ser203. The amino acid sequence is shown above the AlphaFold confidence scores (pLDDT). The color‐coding is according to the AlphaFold confidence scale. The segment surrounding Tyr183 displays moderately increased confidence compared with the predominantly low‐confidence around the Tyr162 region.
**Figure S2:** AlphaMissense prediction of Grb2‐associated‐binding protein 1 (Gab1). Presumably benign mutations are shown in blue and pathogenic mutations in red. Each cell represents a single missense variant with each residue position exchanged against one of the 20 natural amino acids.
**Figure S3:** ^1^H^15^N‐HSQC of the Gab1 fragment. The spectrum was recorded at 298 K, pH 6.5. Assigned amide signals are labeled. Some heterogeneity and weak additional signals are caused by *cis* prolines.
**Figure S4:** NMR intensities of the Gab1 I142‐S203 HCα resonances. The intensities are derived from a ^1^H^13^C ct HSQC.
**Figure S5:** ^15^N R_1_, ^15^N R_2_ and {^1^H‐}^15^N NOE relaxation data.
**Figure S6:** Ramachandran plot of the NMR structure calculation. This plot was created with project on the 10 lowest energy structures.

## Data Availability

The data that support the findings of this study are available from the corresponding author upon reasonable request.
