# Supplementary material for: Inherent α‐Helix Around Tyrosine 183 in the Intrinsically Disordered Tail of the Multi‐Site Docking Platform Gab1
Source: Biopolymers. 2026 Aug 4;117(5):e70120. doi: 10.1002/bip.70120 (PMC13435814; doi:10.1002/bip.70120)
Supplement: Supplementary file 1 [file BIP-117-e70120-s001.docx]

**Inherent α-helix around tyrosine 183 in the intrinsically**

**disordered tail of the multi-site docking platform Gab1**

Anne Dietrich^1^, Marc Lewitzky^1^, Tobias Gruber^1#^, Jochen Balbach^2^*, Stephan M. Feller^1^*, Ulrich Weininger^2^*

^1^Tumor Biology Section, Institute of Molecular Medicine, Martin Luther University Halle-Wittenberg, Halle (Saale), Germany

^2^Biophysics, Institute of Physics, Martin Luther University Halle-Wittenberg, Halle (Saale), Germany

#current affiliation: Navigo Proteins, Halle (Saale)

* Correspondence:

jochen.balbach@physik.uni-halle.de

stephan.feller@uk-halle.de

ulrich.weininger@physik.uni-halle.de

phone: +49 345 55 28555

fax: +49 345 55 27161

**Table S1. Statistics of the NMR structure determination**

| NMR distance and dihedral constraints |  |
| --- | --- |
| Total NOE | 494 |
| intraresidue | 226 |
| sequential (\|i-j\| =1) | 178 |
| medium (\|i-j\| <4) | 90 |
| long (\|i-j\| >5) | 0 |
| Total dihedral angle restraints | 12 |
| phi | 6 |
| psi | 6 |
| Structure statistics |  |
| Violations (mean and SD) |  |
| Distance constraints, Å | 0.03 ± 0.01 |
| Dihedral angle constraints, ° | 0.9 ± 0.3 |
|  |  |
| Ramachandran analysis (all / 180-187) |  |
| Most favored, % | 58.6 / 85.0 |
| Additionally allowed, % | 33.5 / 15.0 |
| Generously allowed, % | 4.7 / 0.0 |
| Disallowed, % | 3.3 / 0.0 |
| Deviations from idealized geometry |  |
| Bonds lengths, Å | 0.003 |
| Bond angles, ° | 0.51 |
| Impropers, ° | 1.25 |
|  |  |

**Figure S1. AlphaFold prediction of the human Grb2-associated-binding protein 1 (Gab1) for the region of Ile142-Ser203.** The amino acid sequence is shown above the AlphaFold confidence scores (pLDDT). The color-coding is according to the AlphaFold confidence scale. The segment surrounding Tyr183 displays moderately increased confidence compared with the predominantly low-confidence around the Tyr162 region.

**Figure S2. AlphaMissense prediction of Grb2-associated-binding protein 1 (Gab1).** Presumably benign mutations are shown in blue and pathogenic mutations in red. Each cell represents a single missense variant with each residue position exchanged against one of the 20 natural amino acids.

**Figure S3. ^1^H^15^N-HSQC of the Gab1 fragment.** The spectrum was recorded at 298 K, pH 6.5. Assigned amide signals are labeled. Some heterogeneity and weak additional signals are caused by *cis* prolines.

**Figure S4. NMR intensities of the Gab1 I142-S203 HCα resonances.** The intensities are derived from a ^1^H^13^C ct HSQC.

**Figure S5. ^15^N R_1_, ^15^N R_2_ and {^1^H-}^15^N NOE relaxation data.**

**Figure S6. Ramachandran plot of the NMR structure calculation.** This plot was created with project on the ten lowest energy structures.
